# Validation of the Focus on the Outcomes of Communication under Six outcome measure

**DOI:** 10.1111/dmcn.12123

**Published:** 2013-03-06

**Authors:** Nancy Thomas-Stonell, Bruce Oddson, Bernadette Robertson, Peter Rosenbaum

**Affiliations:** 1 Bloorview Research InstituteToronto, ON, Canada; 2 University of TorontoToronto, ON, Canada; 3 School of Human Kinetics, Laurentian UniversitySudbury, ON, Canada; 4 CanChild Centre for Childhood Disability Research, McMaster UniversityHamilton, ON, Canada

## Abstract

**Aim:**

The aim of this study was to establish the construct validity of the Focus on the Outcomes of Communication Under Six (FOCUS^©^),a tool designed to measure changes in communication skills in preschool children.

**Method:**

Participating families' children (*n*=97; 68 males, 29 females; mean age 2y 8mo; SD 1.04y, range 10mo–4y 11mo) were recruited through eight Canadian organizations. The children were on a waiting list for speech and language intervention. Parents completed the Ages and Stages Questionnaire – Social/Emotional (ASQ-SE) and the FOCUS three times: at assessment and at the start and end of treatment. A second sample (*n*=28; 16 males 12 females) was recruited from another organization to correlate the FOCUS scores with speech, intelligibility and language measures. Second sample participants ranged in age from 3 years 1 month to 4 years 9 months (mean 3y 11mo; SD 0.41y). At the start and end of treatment, children were videotaped to obtain speech and language samples. Parents and speech–language pathologists (SLPs) independently completed the FOCUS tool. SLPs who were blind to the pre/post order of the videotapes analysed the samples.

**Results:**

The FOCUS measured significantly more change (*p*<0.01) during treatment than during the waiting list period. It demonstrated both convergent and discriminant validity against the ASQ-SE. The FOCUS change corresponded to change measured by a combination of clinical speech and language measures (κ=0.31, *p*<0.05).

**Conclusion:**

The FOCUS shows strong construct validity as a change-detecting instrument.

Evaluating speech and language intervention with validated outcome measures has become increasingly important to the profession of speech and language therapy.^1^,^2^ Outcome measures are tools that inform clinical decision making and provide information that helps clinicians improve services in an evidence-based manner.^3^ Outcome measures are needed to help document the impact of intervention on children's lives.^4^,^5^ Communication impairments can have a broad impact on children's lives, adversely affecting interpersonal interactions, learning, the handling of stress, and other psychosocial demands. Despite a move towards measuring functional outcomes of intervention, there are few measures designed to capture broad communication-related outcomes such as quality of life and social participation.^6^ This limits speech–language pathologists' (SLPs) knowledge about the changes in these domains following intervention.^7^–^9^

Recent literature has urged health professionals to adopt the World Health Organization's International Classification of Functioning, Disability and Health (ICF) and the ICF – Children and Youth (ICF-CY) framework to aid in measuring functional outcomes.^4^,^10^ The American Speech-Language-Hearing Association has adopted the ICF framework[Bibr b11] and in 2010 the Canadian Association of Speech-Language Pathologists and Audiologists identified the need for outcome measures consistent with the ICF and ICF-CY frameworks.[Bibr b12] The components of the ICF-CY include ‘body function and structure’, performance of personal ‘activities’, and ‘participation’ in life situations, as influenced by ‘environmental factors’ and ‘personal factors’.^13^ The ICF-CY provides a conceptual framework for measuring clinical outcomes. To evaluate the full impact of intervention on a child's life, outcome measures must capture the spectrum of changes from individual deficits to life participation.^9^

The Focus on the Outcomes of Communication Under Six (FOCUS)^a^ is a new outcome tool designed for use by either parents or SLPs. It consists of 50 items and takes approximately 10 minutes to complete.^14^ The items were derived from a content analysis of 210 parents' comments (e.g. **‘**My child plays well with other children', ‘My child can communicate independently, ‘My child is understood the first time when she or he is talking with adults who do not know my child well’). The parents described real-world changes in their child's communication skills (e.g. increased socialization, independence, communication intent, and intelligibility) during speech–language therapy.^15^ Their comments aligned with the ICF-CY framework.^16^The FOCUS includes body functions, activities and participation, and personal factors items. It is primarily a measure of activity and performance, with 90% of the items addressing this component. The items measure changes in both capacity and performance.

The FOCUS has strong face validity. The items were selected for reliability and responsiveness and demonstrate high internal consistency (α>0.9) for both the parent and SLP versions.^14^ Interrater and test–retest reliability have been established.^14^ Correlations were found between the FOCUS scores and the Pediatric Quality of Life Inventory, suggesting that the FOCUS measures real-world communication changes that correspond to quality of life.^14^

Treatment outcome measures must be reliable, valid, and responsive to clinically meaningful treatment changes before being adopted into widespread use,^1^ otherwise, professionals may draw incorrect conclusions from the data. Construct validity is an ongoing process of assessing relationships between the measure of interest and other measures or observable phenomena.^16^,^17^ It evaluates whether a measure behaves in a predicted manner during clinical use or whether it demonstrates convergent and discriminant validity with other measures of interest.

The purpose of the study reported here was to assess the construct validity of the FOCUS by exploring three hypotheses.

## Hypothesis 1

The FOCUS will measure more change across a treatment interval than during a waiting list interval. This hypothesis assumes a treatment effect due to speech–language therapy intervention. Research has indicated that speech–language interventions result in treatment effects.^15^,^18^

## Hypothesis 2

The FOCUS will demonstrate convergent and discriminant validity with relevant and non-relevant domains of the Ages and Stages Questionnaire – Social/Emotional (ASQ-SE), a validated screening system which monitors the social and emotional skills of children.^19^

## Hypothesis 3

The FOCUS change scores will demonstrate a moderate relationship with change measured by established speech, intelligibility, and language measures. Previous studies demonstrate that improved speech–language skills at the level of body functions and capacity are associated with improved participation skills.^15^

**What this paper adds**

- The FOCUS is a validated evaluative outcome measure for preschool children receiving speech and language therapy.
- The FOCUS demonstrates both convergent and discriminant validity with the ASQ-SE.
- It detects changes in communication and related participation skills after 9 hours of therapy, primarily provided once a week.

## Method

This study was conducted in two phases. Phase 1 addressed hypotheses 1 and 2. Phase 2 addressed hypothesis 3 using a different sample of children.

Nine partner organizations that provide speech–language services to preschool children in Canada participated. Following ethical approval, SLPs obtained informed consent for study participation from the parents/guardians of children less than 6 years old. Inclusion criteria included children with a speech, language, or communication disorder, identified by registered SLPs, who had been placed on a waiting list for intervention.

### Phase 1

#### Demographic characteristics

A convenience sample of 190 families was recruited from eight of nine partner organizations. Forty families withdrew from speech–language therapy or transferred to another programme and 12 families withdrew from the study because of commitments. There were missing data for 41 families. Complete data were obtained for 97 families. There was no difference in the demographic profile (i.e. age, sex, communication disorder severity) of those children for whom data were missing and those for whom data were complete. Demographic characteristics and variables related to speech–language treatment are described in Table I. On average, children received a total of 9 hours of therapy. Seventy-one per cent of the children received speech–language treatment once per week, consistent with common practice in our partner organizations across Canada. The remaining children received treatment less frequently, ranging from one session every 2 weeks to one session every 3 months. Assessing SLPs completed the communication disorder severity ratings (see Table I) using the Communication Function Classification System, a five-level classification system from level I (most functional) to level V (least functional), developed for children with cerebral palsy.^20^

**Table I:** Characteristics of hypotheses 1 and 2 versus hypothesis 3 samples

Characteristic	Hypotheses 1 and 2 sample (*n*=97)	Hypothesis 3 sample (*n*=28)
Age		
Mean	2.7	3.9
SD	1.04	0.41
Range	0.83–4.9	3.1–4.8
Sex, *n* (%)		
Male	68 (70)	15
Female	29 (30)	13
CFCS, *n* (%)		
Level I	7 (7)	15
Level II	8 (8)	0
Level III	16 (16)	10
Level IV	44 (45)	3
Level V	22 (23)	0
Medical diagnoses, *n* (%)		
Global developmental delay	28 (29)	
Hearing loss	8 (8)	
Syndromes	8 (7)	1
Communication disorder		
Speech and language	81 (84)	28
Language only	8 (8)	
Speech only	8 (8)	
Treatment goal(s)^a^		
Expressive language	71 (73)	25
Receptive language	44 (45)	
Articulation/phonology	39 (40)	28
Amount of treatment (h)		
Mean	8.6	7.1
SD	6.6	4.7
Range	1–46	3–18
Treatment type^a^		
Individual	50 (51)	13
Home programming/consultation	29 (30)	10
Group	25 (26)	7
Parent training	10 (10)(*n*=10)	

Medical diagnoses – top three reported. There was only one medical diagnosis for the hypothesis 3 sample.

a For these categories, percentages add up to more than 100% because some participants have more than one treatment goal and treatment type. CFCS, Communication Function Classification System.

#### Procedures

Parents completed the ASQ-SE and the FOCUS three times: at assessment (time 1), at the start of treatment (time 2), and at end of treatment (time 3). On average, 60 days elapsed between time 1 and time 2, and 90 days between time 2 and time 3. Some communication changes were expected during the waiting list period resulting from communication strategies provided by SLPs. However, our previous research suggested that significantly more change would occur during the treatment period, when children were receiving speech–language intervention.^16^

The ASQ-SE defines social–emotional skills as ‘the array of behaviours that permit one to develop and engage in positive interactions with peers, siblings, parents and other adults, with the ability to regulate emotions effectively and accomplish goals'.^19^ The ASQ-SE evaluates skills in adaptive functioning, self-regulation, autonomy, compliance, communication, affect, and interaction with people. There are eight different forms for children from the age of 6 months to 5 years. Each form addresses all of the domains listed; however, items on each form vary according to the age of the child. The ASQ-SE measures a broader range of social–emotional skills than the FOCUS; however, the FOCUS items measure communicative interactions in social contexts as well as changes in frustration, behaviour, and confidence. Before analysis, ASQ-SE items were coded into two categories: communication and non-communication items. Items that related to communication skills were found in several ASQ-SE domains (e.g. the item ‘follows simple directions’ is in the compliance domain).

#### Analyses

The parents' FOCUS and ASQ-SE change scores during the waiting list and treatment periods were examined using *t*-tests. The relationship between the FOCUS and individual ASQ–SE domains was evaluated using Pearson's rank correlation coefficients. Parent FOCUS scores were selected as the ASQ-SE is also a parent-report measure. Pearson's correlations examined the relationship between FOCUS scores and communication- and non-communication-related items.

The absolute agreement between parent and SLP FOCUS scores at each time point was measured using a two-way random intraclass correlation coefficient (ICC). In addition, parent and SLP descriptive comments were examined to determine whether or not they agreed that functional improvements in communication skills had occurred. A ‘minimal clinically important difference’ (MCID) is defined as minimal changes in the child's function that are considered to be important to both the clinician and parent.^21^ We established a change of greater than or equal to 16 FOCUS points as a conservative estimate of MCID, as the descriptive comments indicated greater than 95% agreement between SLPs and parents that important functional changes had occurred at this level. Kappa was used to examine the level of agreement between parents and SLPs that the FOCUS measured a MCID (i.e. a change of ≥16 points).

### Phase 2

#### Demographic characteristics

Following ethical approval, SLPs from a separate organization obtained informed consent from parents/guardians who had children between the ages of 3 and 6 years. This age range corresponded with the recommended ages for the speech and language measures. Selection criteria included a preschool child who was (1) identified by a registered SLP as having both expressive language and articulation/phonological disorders, and (2) recommended to receive speech–language treatment. Thirty-three children and their families were recruited for the study. Four children were excluded because of technical difficulties with the audio/video recordings. Complete data were collected on 28 of the 29 children. Demographic characteristics and treatment-related variables are described in Table I. The duration of weekly treatment sessions varied from 30 minutes to 1 hour.

#### Procedures

At the start and end of a block of treatment, parents and the SLPs independently completed the FOCUS and the child participated in a videotaped session. The children completed the Children's Speech Intelligibility Measure, imitated phonetically balanced sentences corresponding to a children's story for a Percent Consonant Correct-Revised (PCC-R) analysis, and participated in a free-play session to provide a language sample. An SLP who was unfamiliar with the child and blind to the pre/post order of the videotapes scored the Children's Speech Intelligibility Measure, the PCC-R articulation analysis, and the Developmental Sentence Scoring.

These measures evaluated treatment progress in expressive language, articulation, and/or speech intelligibility for children with communication difficulties of a wide range of severity. The Children's Speech Intelligibility Measure monitors changes in single-word intelligibility during treatment.^22^ It evaluates a variety of phonological competencies while controlling for linguistic variables.^22^ An improvement in single-word intelligibility of greater than or equal to 14% was deemed to be an MCID^22^ based on a combination of effect size and SLPs' observations of functional improvements in intelligibility skills.

PCC-R analysis is one of the best measures of articulation competence for children aged 3–8 years of age and has been extensively researched.^23^ It evaluates phonemic accuracy by determining the frequency of consonant omissions and substitutions. Consonant distortions are scored as correct. Using the same criteria as above, a change greater than or equal to 5% in total scores was deemed to be an MCID.^22^

Developmental Sentence Scoring is a clinical procedure for estimating the status and progress of children's expressive language skills during free-play activities.^24^ Language use during free play is more indicative of communicative-participation skills measured by the FOCUS than most standardized language tests. Progress is evaluated by comparing the child's rate of development at different ages with that of typically developing children. The scores were prorated by the length of the treatment period.^24^ For example, a child who had improved by 0.50 in 1 month was judged to have made an MCID, as the average progress for typically developing children was 0.13 per month (i.e. 0.76 change over a 6mo interval). Lee and Canter^24^ suggest that a child who is progressing at a faster than normal rate during therapy can be deemed to be making good progress.

#### Analyses

Each measure was evaluated separately to determine whether or not the child had met the MCID according to the criteria. If a child met the MCID criteria for any one of the three measures (yes/no), a corresponding MCID was expected on the FOCUS scores (i.e. ≥16 points). Since the FOCUS is a broad measure of communicative competence, it would reflect the combined improvements in articulation, intelligibility, and/or syntax skills. The level of agreement between the communication measures and the FOCUS scores was evaluated using kappa.^25^ Pearson's correlations investigated the relationship between change measured by each speech and language measure and that detected by the FOCUS.

## Results

### Results for hypothesis 1

The FOCUS measured a mean of 5.87 points of positive change during the waiting list period. This change was statistically significant (*t*=2.38; *p*=0.019) but less than the 16 points of change estimated to be required for an MCID. The FOCUS measured a mean of 18.2 points of positive change during the treatment period (*t*=5.62;*p*<0.001). Significantly more change was measured during the speech-language treatment period than the waiting list period (*p*<0.01).

Intraclass correlations examined parent and SLP FOCUS scores. Agreement was high at all three time points (time 1, ICC=0.78; time 2, ICC=0.78; time 3, ICC=0.85; *n*=88). There was fair agreement between parents and SLPs about whether an MCID change (≥16 points) had occurred.^25^ This agreement was significantly better than chance for both the waiting period interval (κ=0.32;*p*<0.01) and the treatment period (κ=0.21; *p*=0.05).

### Results for hypothesis 2

The ASQ-SE total scores did not change significantly during the waiting list period; however, they improved significantly during the treatment period (*t*=3.44; *p*=0.001). The FOCUS change scores correlated significantly with change measured by the ASQ-SE communication questions (*r*=0.232; *p*=0.016; *n*=97), demonstrating convergent validity. The FOCUS change scores did not correlate with change scores from the non-communication items (*r*=0.175; *p*=0.088; *n*=97), demonstrating discriminant validity.

Moderate correlations were obtained between the FOCUS and ASQ-SE scores from the communication, compliance, and affect domains (see Table II).

**Table II:** Correlations between ASQ-SE domains and FOCUS scores

ASQ-SE domains	FOCUS correlations	*p*
Communication	*r*=0.40	0.001
Affect	*r*=0.29	0.02
Compliance	*r*=0.21	0.02
Adaptive functioning	*r*=0.19	0.06
Interaction with people	*r*=0.13	0.22
Autonomy	*r*=0.12	0.27
Self-regulation	*r*=0.02	0.86

ASQ-SE, Ages and Stages Questionnaire – Social/Emotional; FOCUS, Focus on the Outcomes of Communication Under Six.

### Results for hypothesis 3

The FOCUS and the combined speech and language measures agreed that change had occurred in 21 of the 28 children (see Table III). This value represents a fair level of agreement and is significantly better than chance (κ=0.31; *p*<0.05).^25^ The FOCUS change did not correlate with change measured by any of the speech and language measures alone. For three of the seven disagreements, the FOCUS measured clinically significant change that was not captured by the speech and language measures. The other four disagreements occurred because the FOCUS did not measure change or measured negative change despite improvements in the speech and language measures.

**Table III:** Agreements on the minimal clinically important difference between the FOCUS and speech and language measures

	FOCUS		
	
Speech and language measures	Yes	No	Total
Yes	17	4	21
No	3	4	7
Total	20	8	28

FOCUS, Focus on the Outcomes of Communication Under Six.

## Discussion

Although the FOCUS scores significantly improved over both the waiting and the speech–language treatment periods, the FOCUS measured significantly more change during the treatment period. Some change was expected during the waiting list period since general communication strategies were provided; however, more change was expected during the treatment period. SLPs provided therapy to improve specific speech–language skills and provided intensive parent training. The purpose of this hypothesis was to determine whether the FOCUS could detect the expected increase in the amount of change during the treatment period. The increased change may be due to a combination of treatment effects and maturation (as the treatment period was longer than the waiting list period); however, the FOCUS was sensitive to the expected difference in improvement.

Parents and SLPs did not score identical amounts of change on the FOCUS. Generally, SLPs scored less change than parents. SLPs may be more conservative than parents, or differences could be due to parents having more opportunities to see communication changes in different environments. Using the guideline of a 16-point change in the FOCUS scores reflecting an MCID, however, parents and SLPs did agree about whether or not the child had achieved a clinically significant improvement in communication skills.

The ASQ-SE contains many questions that are not related to communication skills and would not be expected to change during speech–language therapy (e.g. ‘Is constipated/has diarrhoea’); therefore, the questions were divided into communication- and non-communication-related items before analysis. As predicted, change measured by the FOCUS correlated with change measured by the ASQ-SE communication questions. There was little change in the ASQ-SE non-communication items scores and no correlation with change measured by the FOCUS_._

Moderate correlations were predicted between the FOCUS and ASQ-SE scores from the domains assessing communication, interaction with people, compliance, and affect. The ASQ-SE measures social–emotional skills (i.e. regulating emotions, accomplishing goals, and engaging in social interactions – both verbal and non-verbal) whereas the FOCUS examines the ability to communicate knowledge, ideas, and feelings in a variety of life settings. Parents' comments about their children reflect the association between social–emotional and communicative participation skills. For example, ‘Socialization is compromised for lack of verbal skills. All behaviours are due to poor communication’; ‘He is not learning words [and] not able to communicate what he wants. Very frustrating for him – tantrums’.

The FOCUS correlated with ASQ-SE scores from the communication, compliance, and affect domains but not with the interaction with people domain, although a correlation was predicted as this domain contained several play questions. The play questions are only included on the 36-, 48-, and 60-month forms. As the mean age of the children in our sample was only 2 years 7 months, the play questions were not applicable to the majority of our sample. The forms for the younger children (<36mo) also contained a higher proportion of non-verbal questions (e.g.. ‘looks for you/is too friendly with strangers’), which would not be responsive to speech–language therapy.

The FOCUS agreed with the combined speech and language measures 75% of the time. Since the FOCUS primarily measures improved communicative participation, it was not expected to correlate with any of the speech and language measures individually. There were also discrepancies between the speech measures on which children had improved. The PCC-R captured improvement in 11 children. The Children's Speech Intelligibility Measure captured improvements in nine children; however, only three of these children showed improvement on both measures.

Most importantly, the disagreements between the FOCUS and the speech–language measures on whether or not an MCID had occurred can be explained. In three children, positive changes in the FOCUS scores, but no improvement on the speech and language measures, were recorded. Parents and SLPs provided comments describing how the children improved during therapy. They indicated that the children had improved in confidence, social skills/friendships, and use of repair strategies. The FOCUS measures changes in these areas. The speech and language measures do not evaluate these areas.

Articulation scores improved significantly in two of the children, but the FOCUS scores improved by less than the 16 points required for an MCID. Parents' and SLPs' comments indicated that the improved articulation skills occurred only when the child was cued by the SLP or parent and had not yet been generalized to conversational speech.

In two children, negative changes were recorded on the FOCUS, despite improvements on the speech and language measures. One child became more disfluent, which adversely affected his willingness to talk to others. The other child started school. The comments indicated that the child was shy and overwhelmed by the new environment and that he was not yet socializing with other children.

One limitation of this study is that parents and SLPs were not blind to the waiting list and treatment periods. Although parents and SLPs independently rated positive changes on the FOCUS and on the ASQ-SE, they may have been biased towards observing changes. It should be noted, however, that ASQ-SE items not related to communication skills did not show change. The reported kappa statistic represents fair agreement. Kappa corrects for chance agreement, and our results simply indicate that agreement between the FOCUS and the speech–language measures was better than could be expected by chance. The Communication Function Classification System, used to classify communication severity, was validated for children between 2 and 18 years of age. Some of the children in our sample (<20%) were younger than 2 years of age.

## Conclusion

The FOCUS shows strong evidence of construct validity with all three hypotheses confirmed. The FOCUS measured significantly more change during the treatment than during the waiting list period. It demonstrated both convergent and discriminant validity with communication-related and unrelated ASQ-SE domains. The FOCUS change scores were correlated with change scores measured by the ASQ-SE communication questions. The FOCUS change scores agreed with changes measured by a combination of speech (Children's Speech Intelligibility Measure/PCC-R) and language (Developmental Sentence Scoring) measures.

The results show that the FOCUS is able to measure changes in communicative competence after an average of 9 hours of speech–language therapy. In addition to improved speech and language skills, the FOCUS measured improvements in confidence, social skills/friendships, and use of repair strategies. An outcome measure that focuses solely on speech and language skills would not measure these important changes. The results indicate that the FOCUS is a valid measure of changes in communication and participation skills for preschool children.

## Footnotes

a The FOCUS is available at http://www.focusoutcomemeasurement.com

## Abbreviations

ASQ-SE: Ages and Stages Questionnaire – Social/Emotional
FOCUS: Focus on the Outcomes of Communication Under Six
ICF: International Classification of Functioning, Disability and Health
ICF-CY: International Classification of Functioning, Disability and Health – Children and Youth
MCID: Minimal clinically important difference
PCC-R: Percent Consonant Correct –Revised
SLP: Speech–language pathologist
